# Prevalence of human papillomavirus in the saliva of sexually active women with cervical intraepithelial neoplasias

**DOI:** 10.4317/medoral.23300

**Published:** 2020-01-22

**Authors:** Mariano Sánchez-Siles, Manuel Remezal-Solano, Antonia María López-López, Fabio Camacho-Alonso

**Affiliations:** 1 DDS, PhD in private oral surgery and medical practice, Murcia. Spain; 2 MD, PhD. Obstetrics and Gynecology, Catholic University of Murcia, Murcia. Spain; 3 MD. Obstetrics and Gynecology, Santa Lucía Hospital, Cartagena. Spain; 4 DDS, PhD. Department of Oral Surgery, University of Murcia, Murcia. Spain.

## Abstract

**Background:**

The main objective of this study was to estimate the prevalence of human papillomavirus-DNA (HPV-DNA) in the saliva of sexually active women with HPV-related cervical intraepithelial neoplasias (CIN) and compare the findings with a healthy control group. The secondary objectives were: 1) to determine the concordance between genital and oral HPV types in sexually active women with HPV-related CIN; 2) to analyze whether sexual habits influence the presence of HPV-related CIN; 3) to determine whether sexual habits influence the presence of oral HPV.

**Material and Methods:**

Saliva samples were collected from 100 sexually active women, 50 with HPV-related CIN and 50 healthy subjects presenting normal cytology. PCR assay was used to detect HPV-DNA.

**Results:**

The prevalence of oral HPV infection in saliva samples was 14% in women with HPV-related CIN, while in the healthy group it was 12%, without statistically significant difference (*p *=0.766). As for the concordance between genital and oral HPV types in women with HPV-related CIN, concordance was only observed for HPV-16, whereby among 22 women with genital HPV-16, only one (4.54%) also presented oral HPV-16. Regarding the possible influence of sexual habits on the presence of cervical pathology and presence of oral HPV, it was found that marital status, age at first intercourse, number of lifetime sexual partners, and condom use are related with the presence of cervical pathology (*p*<0.001; *p*=0.017; *p*=0.002; and *p *<0.001, respectively); condom use was also found to be related to the presence of oral HPV (*p*<0.001).

**Conclusions:**

The prevalence of HPV-DNA in the saliva of sexually active women with HPV-related CIN is similar to healthy women. The concordance between genital and oral HPV types is low. Both the presence of cervical pathology and the presence of oral HPV are related to sexual habits. Wider sample size is required to confirm this results.

** Key words:**Cervical intraepithelial neoplasias, HPV, saliva, cervix, cancer.

## Introduction

Cervical cancer is the second most common cancer among women around the world, and is more frequent (>85% of cases) in developing countries (mainly Asia, Africa, and South America) (1). It is the seventh cause of cancer-related death in Europe. Higher rates occur in Eastern Europe, while lower rates are found in Southern Europe and the Nordic countries. Spain is among the populations at low/moderate risk of cervical cancer, with an incidence rate of 7.1 cases per 100.000 women per year, diagnosing 1.948 cases and recording 712 deaths annually from this type of cancer (2). Infection by human papillomavirus (HPV) has been recognized as an etiological factor for developing cervical carcinoma (3). HPV is a small DNA virus with a circular double-stranded chain of 7,900 base-pairs. The viral particles are proteinic and non-enveloped, very sTable under adverse conditions in an external medium with a capacity for enduring infection. It has a diameter of 55-60 nm, with a viral capsid consisting of 72 capsomers in icosahedral arrangement. The virus is able to infect many regions of the human body, generating multiple clinical manifestations that range from benign hyperplasic lesions to neoplasias of the anogenital region, skin, oral cavity, and pharynx (4). Currently, it is believed that there are more than 150 different types of HPV in existence (with distinct variations and subtypes), and depending on the oncogenic capacity of each type (capacity to establish persistent infection, and promote cell proliferation, altering the DNA of host cells), they are classified by type as: high oncogenic risk [16, 18, 31, 33, 35, 39, 45, 51, 52, 56, 58 and 59]; probable high risk [26, 53, 66, 68, 73 and 82]; and low risk [6, 11, 13, 40, 42, 43, 44, 54, 61, 70, 72, 81, 89, and CP6108] (5). Approximately a third of these are able to infect the epithelium of the genital tract and have been identified as a definitive human carcinogen for six types of cancer: of the cervix, penis, vulva, vagina, anus, and oropharynx (including the base of the tongue and tonsils) (6).

HPV infection of the cervix, is usually related to the appearance of different degrees of cervical intraepithelial neoplasias (CIN), which range from CIN I lesions (slight dysplasia: 1/3 of the cervical cells are abnormal) that retain the capacity to maintain a complete life cycle with virion release; CIN II (moderate dysplasia: 2/3 of cells are abnormal); and CIN III (severe dysplasia/carcinoma in situ: almost all the cervical cells are abnormal). The two latter classifications produce an increase in the expression of two proteins with oncogenic capacity: the E6 and E7 (viral proteins that favor DNA cell attachment); these facilitate the accumulation of mutations in the cell genome, which allows the cancer to progress (7). Nevertheless, although infection of the cervix by HPV is relatively common (up to 80%), cancer is rare (<1%) (8).

In general, infection by HPV among young sexually active women is frequent, but clears up in 80% of cases due to effective immune responses, mediated by CD4+ Th1 response. HPV has various immune evasion systems, as its life cycle is exclusively epithelial and there are no viraemic phases, no cell destruction, and viral replication and virion release is not accompanied by inflammatory processes. Moreover, virion-producing cells that express large amounts of viral antigens are located on the surface of the epithelium away from the immune cells, which are also scarce in the cervical area (9). The key event for progress towards neoplasia could be the deregulation of transforming viral protein expression (E6 and E7), which would lead to increased cell proliferation in lower epithelial layers and an incapacity to repair secondary mutations in host cell DNA. CIN III generally progresses to cancer in lesions that contain integrated copies of the viral genome, in which E7 expression is high. In these lesions with high E6 oncogenic activity, degradation of the p53 tumor suppressor gene is produced, inducing the expression of cyclin-dependent kinase inhibitors p16 (p16INK4a), p21 and p27 (10).

Regardless of whether or not HPV clears up by cervical auto-immune response, sexual behavior has changed in recent decades, the age of sexual initiation has decreased, and lifetime numbers of sexual partners have increased. There has also been a rise in the frequency of oral-to-genital sex, which, in the U.S.A. is more common among Whites (75%), followed by Hispanics (63%), and Afro-Americans (62%) (11). These changes in sexual habits have increased the risk of infection by HPV, not only genitally but also in the oral cavity with particular tropism in the lymphoid tissues, especially the epithelium coating the tonsillar crypts and the base of the tongue that form part of the Waldeyer ring. In fact, there are certain similarities to the cervix, as both proceed from the embryonic endoderm and are epithelia with many invaginations, which could favor antigen capture and processing, facilitating viral access to basal cells.

Over 650.000 patients are diagnosed with head and neck squamous cell carcinoma (HNSCC) worldwide every year and over 350,000 die from it (12). Increased incidence of oropharyngeal carcinoma in recent years, specifically in the amigdala and the base of the tongue (13), in addition to the fact that research has linked HSCNN to HPV, especially HPV-16 (14,15), might suggest that sexually active women with HPV-related genital lesions are at greater risk of presenting some type of HPV in the oral cavity.

The main aim of this study was to estimate the prevalence of HPV-DNA in the saliva of sexually active women with HPV-related CIN compared with a healthy group. The secondary objectives were: 1) to determine the concordance between genital and oral HPV types in sexually active women with HPV-related CIN; 2) to analyze whether sexual habits influence the presence of HPV-related CIN; and 3) to determine whether sexual habits influence the presence of oral HPV.

## Material and Methods

- Recruitment and patient characteristics

The study protocol was approved by the University of Murcia Ethics Committee and was carried out between May 2015 and February 2018 at the Obstetrics and Gynecology Unit, Santa Lucía University Hospital, Cartagena (Spain). Subjects were treated according to guidelines established by the Declaration of Helsinki for medical research involving human subjects. All subjects provided their informed consent to participate. Subjects were recruited applying inclusion criteria recorded in the hospital database: women with and without HPV-related CIN. The entire protocol was carried out by a single clinician.

Inclusion criteria in the study group were as follows: patients aged over 18 years, sexually active women with HPV-related CIN, patients willing to provide informed consent to take part in the study.

For the control group the inclusion criteria were: patients aged over 18 years, sexually active women without HPV-related CIN, patients willing to provide informed consent to take part in the study.

None of the patients who fulfilled the inclusion criteria and were invited to take part in the trial refused to do so. To calculate a representative sample size, a power of 80% was required (5% alpha level). A total of 50 sexually active women with HPV-related CIN and 50 sexually active women without HPV-related CIN were included in this transversal clinical study.

Having received their informed consent in writing, a detailed anonymous questionnaire was administered in order to collect information about age, smoking, drinking, and sexual habits.

- Sample collection and HPV-DNA detection

All saliva samples (n=100) were collected under equal conditions (first thing in the morning, instructing subjects not to eat or drink for 90 minutes before sample collection). Patients were placed in a relaxed position and looking downwards. Patients were asked to rinse the mouth vigorously and gargle with 10 ml normal saline for a total of 30 seconds (10 seconds rinse, 5 seconds gargle, 10 seconds rinse, and 5 seconds gargle). Specimens were collected in sterile tubes and refrigerated to minimize degradation of salivary proteins until further processing. The saliva samples were centrifuged at 2000 rpm at 4ºC for 10 min, 2 ml supernatant saved, followed by re-suspension of the cell pellet in 10 ml PBS, which was centrifuged at 2000 rpm at 4ºC for 10 min, then re-suspended in 3 ml PBS, and split into two aliquots. All samples were stored locally at -80ºC until DNA purification, amplification, and HPV-DNA detection.

All samples were analyzed by the same geneticist in a blinded fashion. DNA was purified from all samples by use of a Puregene DNA Purification Kit (Gentra Systems, Minneapolis, Minnesota, USA) (16). The presence of HPV genomic DNA in saliva samples was detected by polymerase chain reaction (PCR) amplification using the PGMY09/11 L1 consensus primer system (17); the type was specified by hybridization to an HPV probe array containing 32 HPV types (high oncogenic risk: 16, 18, 31, 33, 35, 39, 45, 51, 52, 56, 58 and 59; probable high risk: 26, 53, 66, 68, 73 and 82; and low risk: 6, 11, 13, 40, 42, 43, 44, 54, 61, 70, 72, 81, 89, and CP6108) (6), and β-globin (Roche Molecular Systems, Alameda, California, USA) (18).

- Statistical analysis

Data were analyzed using the SPSS 20.0 statistics program (SPSS® Inc, Chicago, IL, USA). A descriptive study was made of each variable. The Kolmogorov-Smirnov normality test and Levene's homogeneity of variance test were applied; the data showed normal distribution and so were analyzed using parametric tests. Associations between the different qualitative variables were studied using Pearson’s chi-squared test. Associations between different quantitative variables were studied using Student’s t-test for two related samples. Adjusted odds ratios and confidence intervals were calculated using multiple logistic regression models considering the binaries “presence of HPV-related CIN” and “presence of oral HPV” as the outcome variables, and using marital status, age at first intercourse, number of lifetime sexual partners, oral-to-genital sex, and condom use as covariates. Statistical significance was accepted for p≤0.05.

## Results

A total of 100 sexually active women, 50 with HPV-related CIN (with a mean age of 34.54 ± 7.66) and 50 healthy control subjects without HPV-related CIN were included in this transversal clinical study. Mean patient age was 36.92 ± 6.69. Both groups were homogenous in terms of age (*p*=0.101), educational level (*p*=0.505), smoking (*p*=0.683), and alcohol consumption (*p*=0.517). Nevertheless, a higher percentage of women with HPV-related CIN were single or not in a sTable couple relationship (88% compared with 34% of control subjects), had had >4 children (16% compared with 4%), had had their first sexual intercourse aged ≤16 years (30% compared with 10%), had had a number of lifetime sexual partners ≥2 (98% compared with 60%), practiced oral-to-genital sex (92% compared with 70%), had had some sexually transmitted infection (16% compared with 4%), and a lower percentage of women in this group used condoms (34% compared with 74%); with statistically significant differences (*p*<0.001; *p*=0.046; *p*=0.004; *p*=0.001; *p*=0.005; *p*=0.046; and *p*=0.001, respectively) (Table 1).

**Table 1:**
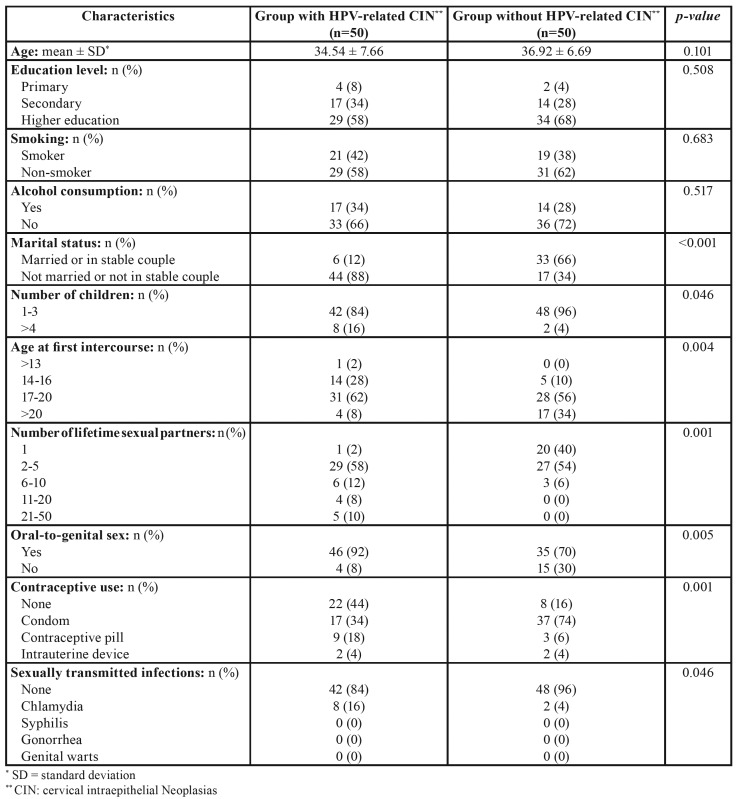
Comparison of demographic characteristics in study groups: educational level, sexual history and habits between study groups (Student t-test and Pearson χ2).

The prevalence of HPV-DNA in saliva was found to be similar between the two groups, 14% in women with HPV-related CIN compared with 12% in women without HPV-related CIN, with no statistically significant difference (*p*=0.766). Only two (4%) women in the group with HPV-related CIN presented more than one type of oral HPV (two types) and only one women (2%) in the healthy control group, without statistically significant difference (*p*=0.842). When oral HPV types were compared between groups, nine different types were diagnosed in seven women with HPV-related CIN and HPV-DNA in saliva, the most common type being 45 but no significant difference was found in comparison with the control group (33.33% compared with 14.28%; *p*=0.308). In the same way, it was observed that of the seven types diagnosed in six of the women without HPV-related CIN and HPV-DNA in saliva, the most frequently occurring type was 39 but without significant difference in comparison with its presence in women with HPV-related CIN (28.57% compared with 22.22%; *p*=0.853) (Table 2).

**Table 2:**
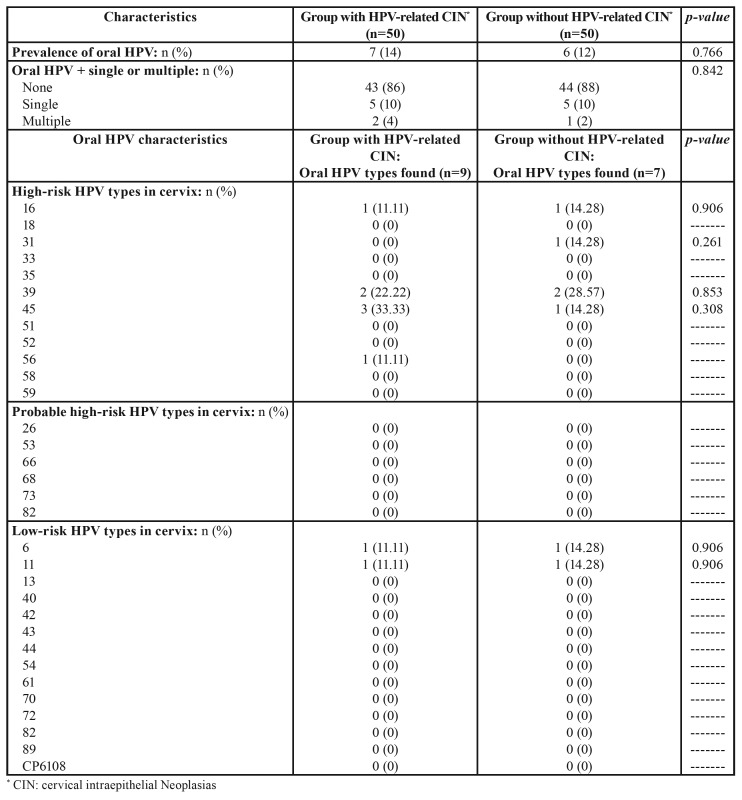
Comparison of oral HPV characteristics between groups (Pearson χ2 test).

Regarding the characteristics of genital HPV in women with HPV-related CIN, 50% presented multiple types, making a total of 93 types, the most common being type 16 (23.65%) (Table 3). When the concordance of genital and oral HPV types in group with HPV-related CIN was analyzed, among 22 women with genital HPV-16, only one (4.54%) also presented oral HPV-16; for the remaining 92 types genital HPV types, no concordance was obtained between cervical and oral HPV types (Table 4).

**Table 3:**
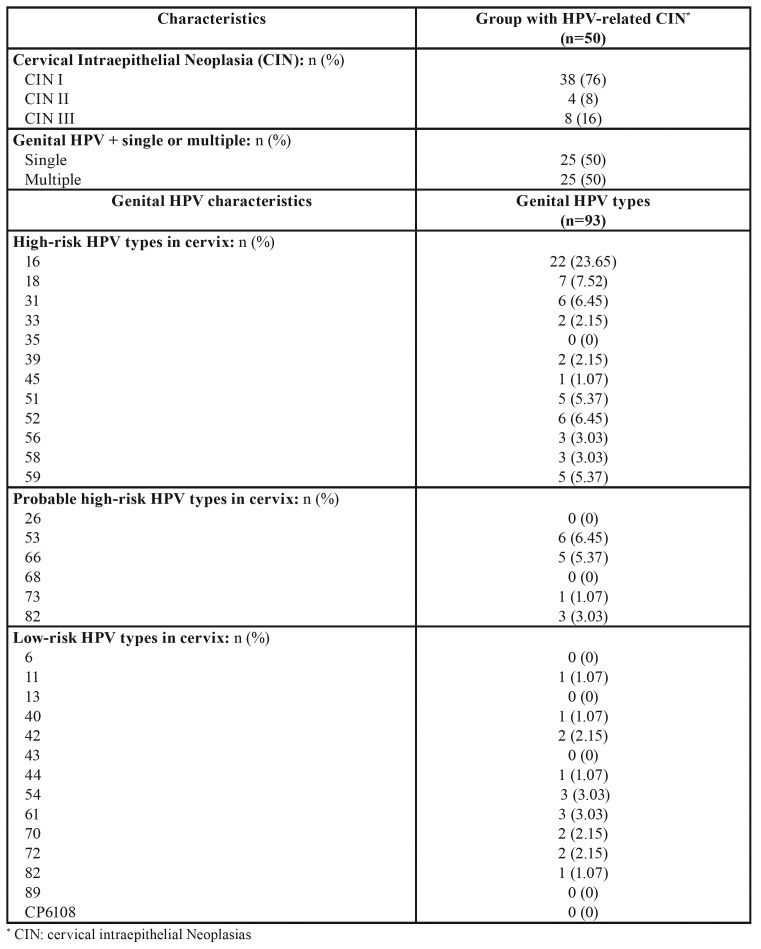
Description of cervical intraepithelial neoplasia and genital HPV characteristics in group with HPV-related CIN.

**Table 4:**
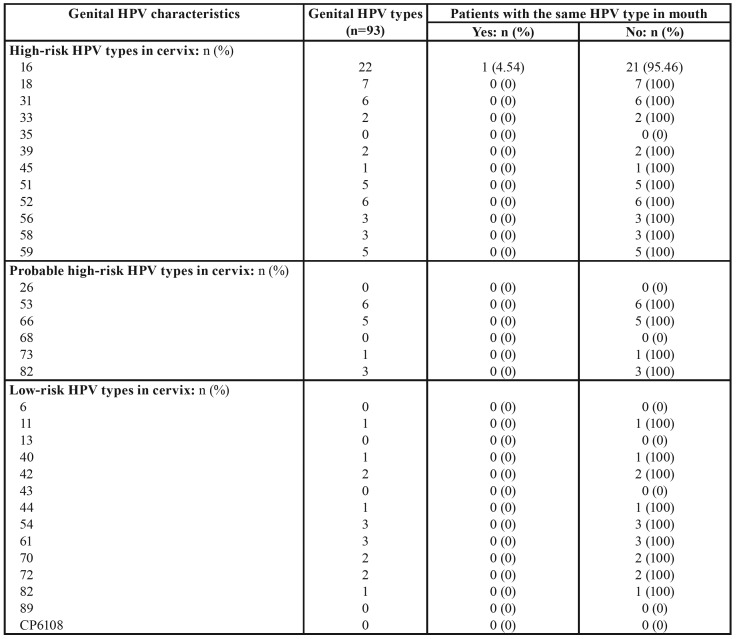
Concordance of genital and oral HPV types in group with HPV-related CIN.

When sexual habits were analyzed to determine whether these exert any influence on the presence of HPV-related CIN, it was found that marital status, age at first intercourse, number of lifetime sexual partners, and condom use were all related to presence of HPV-related CIN (OR: 0.07; 3.85; 0.20; and 5.52 respectively) (95% CI: 0.02-0.19; 1.27-11.63; 0.07-0.56; and 2.33-13.07 respectively) (*p*<0.001; *p*=0.017; *p*=0.002; and *p*<0.001 respectively).

Lastly, when sexual practices (marital status, age at first intercourse, number of lifetime sexual partners, oral-to-genital sex, and condom use) were analyzed to determine whether they had any influence on the presence of oral HPV, only condom use was found to be related to the presence of oral HPV (OR 45.00, 95% CI 9.71-208.59, *p*<0.001).

## Discussion

HPV infection is the most common sexually transmitted disease, which may affect as many as 70% of young women (6). Among women infected with HPV, a small number will go on to develop CIN (I, II, or III), and eventually cancer (8). When cytological screening detects an abnormality, this will be further investigated by means of a colposcopy with targeted cervical biopsy, HPV-DNA analysis, and endocervical curettage if considered necessary. The therapeutic procedures for dealing with CIN range from treatments that destroy tissue (cryoagulation or laser vaporization) to the most commonly used excisional treatment using conization, which consists of the removal of a cone-shaped wedge of tissue from external part of the uteris or exocervix (the base of the cone) and an internal part corresponding to the cervical canal or endocervix (the tip of the cone) (19). Recent decades have seen changes in sexual behaviors, with increased practice of oral-to-genital sex (11), which suggests that women with HPV-related CIN may be at greater risk of presenting some type of HPV in the oral cavity. This possibility, together with the fact that oral HPV is increasingly found to be related to HSCNN (14,15), are the reasons behind the present study’s objectives.

The main aim was to estimate the prevalence of HPV-DNA in the saliva of sexually active women with HPV-related CIN compared with a healthy control group. Prevalence was found to be similar in both groups, 14% in women with HPV-related CIN compared with 12% in women without HPV-related CIN, without statistically significant difference (*p*=0.766). Similar results have been obtained by other authors such as Adamopoulou *et al*., (20) who analyzed saliva samples using PCR of 43 sexually active women without HPV-related CIN; oral HPV prevalence was 11.6%, almost identical to the prevalence found in the present control group of healthy subjects and very similar to the study group of women with HPV-related CIN. But others, such as Siani *et al*. (21), who analyzed saliva samples from 70 sexually active women with cervical cancer, found a very low prevalence of oral HPV (4 out of 70, 5.71%). But other studies such as Visalli *et al*. (22), who, like the present study, investigated the prevalence of HPV in the saliva of 100 women with HPV cervical lesions compared with a healthy control group of 25 women with normal cytology, obtained greater prevalence in women with HPV cervical lesions than the control group (24% compared with 8%), with statistically significant differences (p≤0.05). Likewise, Giraldo *et al*., (23) compared the prevalence of HPV in the oral cavity among 70 women presenting histopathologically confirmed clinical HPV lesions in the genital region and 70 with HPV-related genital lesions, obtaining much higher prevalence among women with HPV lesions in the genital region (37.1% compared with 4.3%), with strongly significant difference (*p*<0.001).

This large discrepancy in results for the possible greater prevalence of oral HPV in sexually active women with HPV-related CIN reported in the literature, could be related to three main aspects: firstly, the differences in the populations studied; secondly, methodological differences between studies; and thirdly, variations in sexual practices (20). Regarding populations, the published literature includes a diversity of populations and specimen types; nevertheless, co-infection of multiple anatomical sites in the same individual can be attributed to factors such as genetic predisposition or inadequate immune response. Any alteration in immunity, whether general or local, can favor the colonization, replication and persistence of HPV in regions such as the oral cavity (24). As for methodology, there is great variability in the screening efficacy of HPV detection methods among the articles published. In this sense, in the study by Adamopoulou *et al*., (20) which obtained a prevalence of oral en HPV in sexually women without HPV-related CIN of 11.6% using PCR, this prevalence increased in the same group to 44.2% with nested PCR. To achieve homogeneity among this type of study, it is important that sexual behaviors that may affect the appearance of co-infection at both anatomic sites (cervix and oral cavity), such as a history of oral-to-genital contact and condom use during this contact, should be recorded in all studies, as these and other sexual practices could increase or decrease the prevalence of oral HPV in sexually active women both with and without HPV- related CIN (25,26). In this context, one of the main limitations of the present work was the small sample size (wider sample size is required to confirm this results), a lack shared with other similar studies. For this reason, more homogeneous studies with bigger sample sizes are needed to determine the relationship between HPV infection in these two areas.

Regarding the present study’s first secondary objective (to determine the concordance between genital and oral types in sexually active women with HPV-related CIN), among 22 women with genital HPV-16, only one (4.54%) also presented oral HPV-16; while for the other 92 HPV genital types, no concordance was found with oral types of HPV. Similar findings have been reported by authors such as Sayyah-Melli *et al*., (27) who genotyped by PCR the HPV-DNA in the saliva of 104 sexually active women with HPV-related genital lesions for types 16, 18, 31, 33 (high risk) and 6, and 11 (low risk), observing greater genital/oral concordance for HPV-16 type, this being higher than in the present study (10.6%). Vogt *et al*., (28) obtained similar results in a study of 37 different types of HPV-DNA in saliva, obtaining a concordance of HPV genital and oral types of 12%. Smith *et al*., (29) investigating the possible concordance between genital and oral HPV types in a total of 577 pregnant women (without HPV-related CIN) found that HPV prevalence was 29% in the cervix and 2.4% in the oral cavity, and that there was no type-specific HPV concordance between these two sites. One explanation for the lack of concordance between the oral and genital sites may be due to non-persistent or intermittent detection of HPV over time, as shown in studies of the cervix (30). This phenomenon of intermittent HPV detection over time has not been studied in relation to the oral cavity. Perhaps oral and genital specimens need to be collected at multiple intervals to detect fluctuating levels of infection in an individual, although it is questionable whether repeat measurements would reduce differences in prevalence rates at the two mucosal sites. Nevertheless, although longitudinal studies could obtain some modification of the low concordance of genital and oral HPV types, it is unclear to what extent differences between the two sites would be reduced (29).

Regarding the present study’s second secondary objective (to determine whether sexual behaviors have an influence on the presence of HPV-related CIN), it was found that marital status, age at first intercourse, number of lifetime sexual partners, and condom use were related to the presence of HPV-related CIN (*p*<0.001; *p*=0.017; *p*=0.002; and *p*<0.001 respectively). Similar results were obtained by authors such as Saini *et al*., (21), who, in their study of 70 sexually active women with cervical cancer, found that 17.1% were not married or not in sTable relationships. Others such as Smith *et al*., (29) in their study of 577 pregnant women (without HPV-related CIN), found an HPV prevalence of 29% in the cervix in a multiple logistic regression model considering the binary “presence of HPV in cervix” as the outcome variable, and using marital status, age at first intercourse, and number of lifetime sexual partners as covariates; they found that these variables related to sexual behavior did exert an influence on “presence of HPV in cervix.” Similarly, Schlecht *et al*., (26), in a study of the influence of sexual behaviors on the presence of genital HPV in a sample of 645 sexually active women, also found that marital status, age at first intercourse, number of lifetime sexual partners, and condom use, were covariates closely related to cervical HPV infection. Both the present results and those of the other authors cited above point to a need for greater social awareness of: multi-type prophylactic HPV vaccines, continued screening strategies to prevent HPV-related diseases, and the need for sexual healthcare information targeted at sexually active women.

Regarding the present study’s third secondary objective (to determine whether sexual behaviors influence the presence of oral HPV), the use or not of condoms was strongly related to the presence of oral HPV (*p*<0.001). Similar results were obtained by Schlecht *et al*., (26), who in their study of the influence of sexual behaviors on the presence of genital and oral HPV among a sample of 645 sexually active women, found that condom use or non-use during oral-to-genital sex were closely related to the presence of oral HPV. In the same way, Ragin *et al*., (25) in their study of the prevalence of oral HPV in 118 sexually active women, found that 66.6% of women with oral HPV practiced oral-to-genital sex (58.3% with men and 8.3% with women) without using a condom, so that non-use of this preventative measure was significantly related to the presence of HPV in the oral cavity (*p*=0.039). The results of the present study analyzed by means of a logistic regression model found that the practice of oral-to-genital sex showed no relation to the presence of oral HPV (*p*=0.288), but the use or non-use of condoms did (*p*<0.001) indicating that oral-to-genital sex does not increase the risk of oral HPV infection providing a condom is used.

In conclusion, this study shows that the prevalence of HPV-DNA in the saliva of sexually active women with HPV-related CIN is similar to that of healthy control subjects and that the concordance between genital and oral HPV types is low, which may indicate that the natural history of oral and cervical infection differs; both the presence of cervical pathology and the presence of oral HPV are related to sexual behaviors. Nevertheless, wider sample size is required to confirm this results.
